# Structural flexibility of halogen bonds showed in a single-crystal-to-single-crystal [2+2] photodimerization

**DOI:** 10.1107/S2052252518007583

**Published:** 2018-06-22

**Authors:** Michael A. Sinnwell, Jared N. Blad, Logan R. Thomas, Leonard R. MacGillivray

**Affiliations:** a Department of Chemistry, University of Iowa, Iowa City, Iowa 52242, USA

**Keywords:** solid-state reactivity, halogen bonds, photodimerization, supramolecular chemistry, single-crystal-to-single-crystal reaction, crystal engineering, co-crystals, organic solid-state reactions, framework-structured solids and amorphous materials, molecular crystals

## Abstract

Halogen bonds have emerged as noncovalent forces that govern the assembly of molecules in organic solids with a degree of reliability akin to hydrogen bonds. Although the structure-directing roles of halogen bonds are often compared to hydrogen bonds, general knowledge concerning the fundamental structural behavior of halogen bonds has had limited opportunity to develop. Following an investigation of solid-state reactions involving organic syntheses and the development of photoresponsive materials, this work demonstrates the ability of the components of intermolecular N⋯I halogen bonding – a ‘workhorse’ interaction for the crystal engineer – to support a single-crystal-to-single-crystal [2+2] photodimerization. A comparison is provided of the geometric changes experienced by the halogen-bonded components in the single-crystal reaction to the current crystal landscape of N⋯I halogen bonds, as derived from the Cambridge Structural Database. Specifically, a linear-to-bent type of deformation of the halogen-bonded components was observed, which is expected to support the development of functional halogen-bonded materials containing molecules that can undergo movements in close-packed crystal environments.

## Abstract 2

Single-crystal-to-single-crystal (SCSC) reactions are rare for organic solids. We describe the first example of an SCSC [2+2] photodimerization that is mediated by halogen bonding.

## 1. Introduction   

Halogen bonds are employed in supramolecular chemistry to direct the assembly of molecules in solution and the solid state (Gilday *et al.*, 2015 ▸, Resnati *et al.*, 2015 ▸). The strength and directionality of halogen bonds parallel that of hydrogen bonds such that halogen bonding provides a reliable force to organize molecules in the construction of functional crystalline solids (Cavallo *et al.*, 2016 ▸) and other condensed materials (*e.g.* liquid crystals, polymers).

A hallmark feature of noncovalent bonding when controlling self-assembly is the structural flexibility of components engaged in intermolecular forces (Desiraju, 2011 ▸). Hydrogen bonds generally exploit the flexibility of *X*—H donor groups (*e.g.* *X* = O, N) to accommodate the orientation and assembly of components into supramolecular structures. Flexibility in the solid state can give rise to atomic and molecular movements that support single-crystal-to-single-crystal (SCSC) transformations (Friščić & MacGillivray, 2005 ▸). Solids that undergo SCSC transitions bear relevance to applications of organic solid-state materials in areas such as data storage, sensors and shape memory (Kole *et al.*, 2014 ▸).

The pyridine–iodo­polyfluorophenyl (N⋯I) halogen bond has been exploited extensively in the field of crystal engineering to sustain discrete and extended supramolecular frameworks (Cavallo *et al.*, 2016 ▸). Computational studies have recently demonstrated that the energies of the N⋯I bond exhibit a relatively high dependence on the C—I⋯N angle θ (*ca* 150° < θ < 180°) (Tsuzuki *et al.*, 2016 ▸). It is not clear whether the components of N⋯I halogen bonds, however, can tolerate types of bending motions similar to hydrogen-bonded components in the solid state, although rotary movements of halogen-bonded molecular components have been recently recognized as important in the development of artificial molecular machines (Catalano *et al.*, 2015 ▸). The bending of components engaged in noncovalent forces (*e.g.* hydrogen bonds, halogen bonds) can be considered fundamentally important in the development of machine-like materials (Khuong *et al.*, 2006 ▸). Examples of halogen-bonded solids that support single-crystal processes, however, remain rare and to date have involved molecules that provide internal degrees of molecular flexibility and freedom (*e.g.* –CH_2_– groups, –N=N–) (Sun *et al.*, 2006 ▸; Luo *et al.*, 2008 ▸; Raatikainen & Rissanen, 2012 ▸; Marti-Rujas *et al.*, 2012 ▸; Jin *et al.*, 2013 ▸; Bushuyev *et al.*, 2013 ▸; Bushuyev *et al.*, 2014 ▸). We are unaware of an SCSC [2+2] photodimerization supported by halogen bonds.

In this contribution, we report a halogen-bonded cocrystal with components that support an SCSC [2+2] photodimerization (Scheme 1). We show the components of (I_4_F_16_cb)·2(bpe) [where I_4_F_16_cb = *rctt*-tetrakis(2,3,5,6-tetrafluoro-4-iodophenyl)cyclo­butane and bpe = *trans*-1,2-bis(4-pyridyl)­ethene], which assemble *via* N⋯I halogen bonds to form a cocrystal that reacts quantitatively to form tpcb (tpcb = *rctt*-tetrakis­(4-pyridyl)­cyclo­butane). We demonstrate that the components engaged in halogen bonding exhibit structural flexibility, their geometries undergoing a linear-to-bent type of deformation in the SCSC transformation. The movements span the outer geometries of N⋯I bonds that have been reported to date.
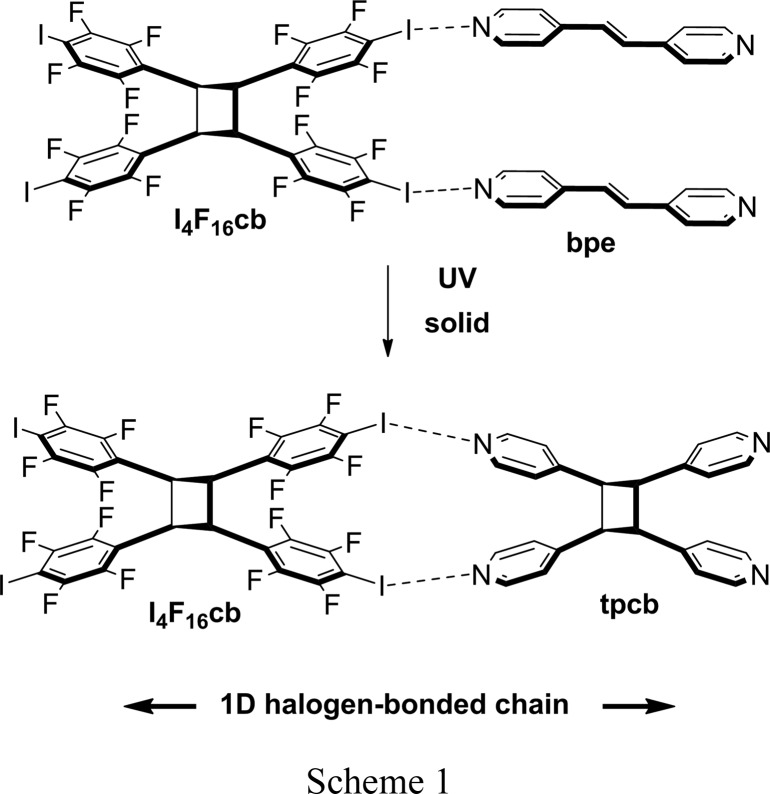


## 2. Experimental   

### 2.1. Materials and general methods   

The bipyridine bpe (SIGMA) and all solvents were commercially available and were used without further purification (FISHER). ^1^H NMR spectra were recorded using a Bruker AVANCE-300 NMR spectrometer operating at 300 MHz using DMSO-*d*_6_ as solvent. Photoreactions of (I_4_F_16_cb)·2(bpe) were conducted on a glass plate using UV radiation from a 450 W medium-pressure mercury lamp inside an ACE Glass photochemistry cabinet. Powder X-ray diffraction data were collected from samples mounted on glass slides by a Siemens D5000 X-ray diffractometer using Cu *K*α_1_ radiation (λ = 1.54056 Å).

### 2.2. Synthesis of cocrystal (I_4_F_16_cb)·2(bpe)   

I_4_F_16_cb was synthesized as reported (Sinnwell & MacGillivray, 2016 ▸). Cocrystals of (I_4_F_16_cb)·2(bpe) were obtained as colorless plates by combining aceto­nitrile solutions (1.5 ml each) of I_4_F_16_cb (23 mg, 0.02 mmol) and bpe (11 mg, 0.04 mmol) (1:2 molar ratio). Single crystals suitable for single-crystal X-ray diffraction studies were obtained after a period of approximately 1 d.

### 2.3. X-ray crystallography   

Single-crystal data for (I_4_F_16_cb)·2(bpe) were collected with a Bruker APEXII Kappa diffractometer equipped with an Oxford Cryostream low-temperature device using Mo *K*α radiation (λ = 0.71073 Å). All calculations dealing with data collection, initial indexing, frame integration, Lorentz polarization corrections and final unit-cell parameters were carried out by *APEX2* (Bruker, 2012 ▸). Single-crystal diffraction data for (I_4_F_16_cb)·2(tpcb) were collected on a Nonius KappaCCD single-crystal X-ray diffractometer at room temperature using Mo *K*α radiation (λ = 0.71073 Å). Data collection, cell refinement and data reduction were performed using *COLLECT* (Hooft, 1998 ▸) and HKL *SCALEPACK/DENZO*, respectively (Otwinowski & Minor, 1997 ▸). All structures were solved *via* direct methods using *SHELXT* (Sheldrick, 2015 ▸) and refined using *SHELXL* (Sheldrick, 2015 ▸) in the *OLEX2* (Dolomanov *et al.*, 2009 ▸) graphical user interface. All non-H atoms were identified from difference Fourier maps within several refinement steps. H atoms associated with C atoms were refined in geometrically constrained positions with *U*_iso_(H) = 1.2*U*_eq_(C).

#### 2.3.1. Crystal data for (I_4_F_16_cb)·2(bpe)   

Crystal system: monoclinic, space group: *P*2_1_/*c* (No. 14), *a* = 13.0184 (13), *b* = 9.7153 (10), *c* = 19.986 (2) Å, *β* = 104.649 (5)°, *V* = 2445.6 (4) Å^3^, *Z* = 2, *T* = 298.15 K, μ(Mo *K*α) = 2.653 mm^−1^, *D*_calc_ = 2.059 g cm^−3^, 52 897 reflections measured (5.926° ≤ 2θ ≤ 52.87°), 5012 unique (*R*_int_ = 0.0305, *R*_sigma_ = 0.0162) which were used in all calculations. The final *R*_1_ was 0.0198 [*I* > 2σ(*I*)] and *wR*_2_ was 0.0460 (all data).

#### 2.3.2. Crystal data for (I_4_F_16_cb)·2(tpcb)   

Crystal system: monoclinic, space group: *P*2_1_/*c* (No. 14), *a* = 12.9647 (13), *b* = 10.2219 (10), *c* = 19.519 (10) Å, *β* = 109.387 (5)°, *V* = 2440.0 (13) Å^3^, *Z* = 2, *T* = 298.15 K, μ(Mo *K*α) = 2.659 mm^−1^, *D*_calc_ = 2.064 g cm^−3^, 12 227 reflections measured (5.956° ≤ 2θ ≤ 50.786°), 4464 unique (*R*_int_ = 0.0670, *R*_sigma_ = 0.0580) which were used in all calculations. The final *R*_1_ was 0.0330 [*I* > 2σ(*I*)] and *wR*_2_ was 0.0627 (all data).

## 3. Results and discussion   

Our interests lie in the design of organic solids using molecules that react to form covalent bonds. The ability to direct the formation of covalent bonds in crystals ‘by design’ can provide a means of synthesizing organic molecules that are difficult or impossible to achieve in solution (*e.g.* ladderanes) and/or of controlling the bulk-physical properties of solids (*e.g.* optical). In this context, a major focus has been the use of noncovalent forces to direct the formation of carbon–carbon single (C—C) bonds in cocrystals *via* [2+2] photodimerizations (MacGillivray *et al.*, 2008 ▸). The formation of covalent bonds in solids is invariably accompanied by atomic and/or molecular movements (Friščić & MacGillivray, 2005 ▸) that often result in crystal cracking and can lead to macroscopic motility in the form of popping (Medishetty *et al.*, 2014 ▸). Cocrystals sustained by hydrogen bonds have been shown in limited cases to support [2+2] photodimerizations that proceed as SCSC transformations (MacGillivray *et al.*, 2008 ▸; Santra & Biradha, 2008 ▸). An SCSC transformation provides the ability to observe subtle, atomic level changes to structures induced by external stimuli (*e.g.* UV radiation) and thus provide insight into the structural behaviors and dynamics of molecular components engaged in intermolecular bonding. Although there have been two recent reports of intermolecular N⋯I halogen bonds that support photo­cyclo­addition reactions in the solid state (Caronna *et al.*, 2004 ▸; Sinnwell & MacGillivray, 2016 ▸), we are unaware of any examples of halogen-bonded solids with components that can undergo an SCSC photodimerization.

### 3.1. Halogen bonds in SCSC photo­dimerization   

The halogen-bonded cocrystal that undergoes SCSC photodimerization is (I_4_F_16_cb)·2(bpe). We have reported the synthesis of I_4_F_16_cb in the solid state using 1,8-bis­(4-pyridyl)­naphthalene (dpn) as a template (Sinnwell & MacGillivray, 2016 ▸). Here we identify I_4_F_16_cb as a rare example of a template that operates by reversal of the noncovalent bonding (Bhattacharya *et al.*, 2013 ▸). Slow evaporation of a solution of I_4_F_16_cb and bpe (1:2 ratio) in aceto­nitrile afforded colorless plate-shaped crystals after a period of 1 d. The formulation of (I_4_F_16_cb)·2(bpe) was confirmed by single-crystal and powder X-ray diffraction (at 298 K), as well as by ^1^H NMR spectroscopy.

**Figure 1:**
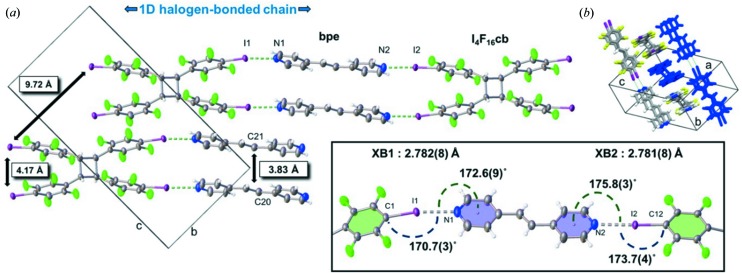
X-ray structure of (I_4_F_16_cb)·2(bpe): (*a*) C=C distances (inset: halogen-bonding metrics) and (*b*) layers of chains.

**Figure 2:**
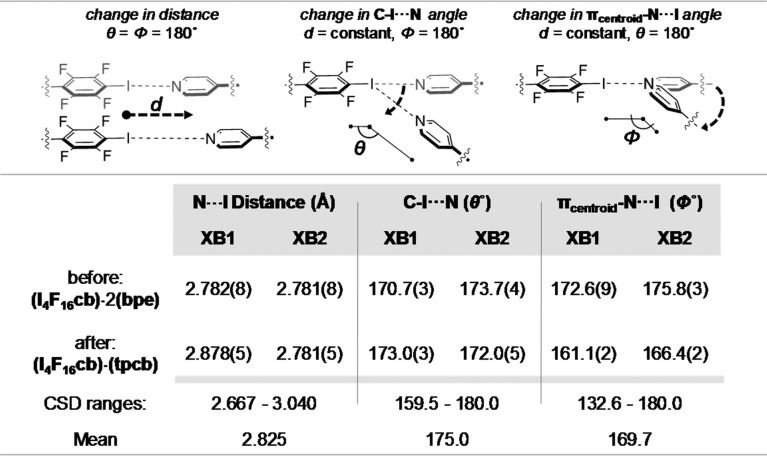
Table of halogen-bond metrics and changes in the geometries of components of (I_4_F_16_cb)·2(bpe) and (I_4_F_16_cb)·(tpcb) with comparisons to the CSD.

Single-crystal X-ray analysis revealed that (I_4_F_16_cb)·2(bpe) crystallizes in the monoclinic space group *P*2_1_/*c*. Half a molecule of I_4_F_16_cb and one molecule of bpe lie in the asymmetric unit (Fig. 1 ▸). The components form one-dimensional chains sustained by two unique N⋯I halogen bonds (*X* *B*1 = N1⋯I1; *X* *B*2 = N2⋯I2) (see the table in Fig. 2 ▸). Cyclo­butane I_4_F_16_cb, which is disordered over two sites [highest occupied site: 0.607 (5)], organizes bpe in a face-to-face π-stacked geometry with two C=C bonds of bpe parallel and separated by 3.83 Å. The olefin lies disordered over two sites [highest occupied site: 0.769 (7)]. The stacking satisfies the criteria for a [2+2] photodimerization (Schmidt, 1971 ▸). The one-dimensional chains lie offset and parallel along the crystallographic *b* axis *via* face-to-face interactions of* p*-C_6_F_4_-I and pyridyl rings [centroid–centroid (π–π): π_F_–π_F_ = 3.40, π_F_–π_pyr_ = 3.99 and π_pyr_–π_pyr_ = 3.84 Å]. Olefins of neighboring chains are separated by 7.89 Å which is outside the limits for a photoreaction.

When single crystals of (I_4_F_16_cb)·2(bpe) were exposed to UV irradiation (450 W medium-pressure Hg lamp) for a period of 30 h, tpcb formed quantitatively, indicated by the disappearance of the olefinic peaks (7.54 p.p.m.) and the appearance of cyclo­butane peaks (4.66 p.p.m.) in the ^1^H NMR spectrum (DMSO-*d*_6_). Optical microscopy confirmed that the crystals remained intact during the photodimerization.

**Figure 3:**
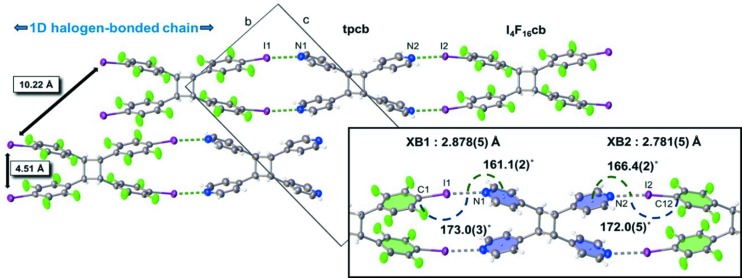
X-ray structure of (I_4_F_16_cb)·(tpcb) showing cyclo­butane formation (inset: halogen-bonding metrics).

An X-ray diffraction analysis (at 298 K) confirmed that (I_4_F_16_cb)·2(bpe) undergoes an SCSC reaction to form (I_4_F_16_cb)·2(tbcb). The bpe olefin reacted to give tpcb and, as a result, maintained the structure of the halogen-bonded chains (*XB*1 = N1⋯I1; *XB*2 = N2⋯I2) (Fig. 3 ▸). The halogenated cyclo­butane of the photoreacted cocrystal adopts two orientations [highest occupied site: 0.764 (7)] with site occupancies that differ from I_4_F_16_cb of the unreacted solid. A comparison of the unit-cell dimensions revealed that the volume and density remained virtually unchanged. A calculated 5% increase in the *b* axis relates to a change in conformation of I_4_F_16_cb manifested by a splaying of the pendant *p*-C_6_F_4_-I groups. The splaying accommodates newly formed tpcb whilst maintaining the halogen bonds.

### 3.2. Crystal landscape of halogen bonds   

**Figure 4:**
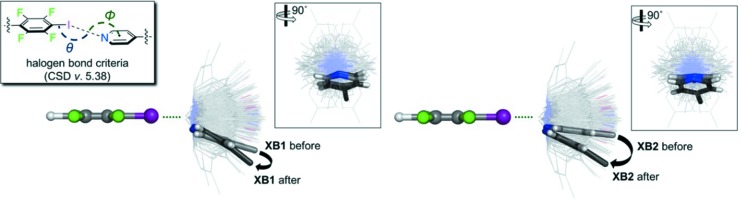
*IsoStar* scatterplots: *XB*1 (left) and *XB*2 (right) of (I_4_F_16_cb)·2(bpe) (gray) and (I_4_F_16_cb)·(tpcb) (black). Highest occupied *XB*1 and *XB*2 in one quadrant (inset: criteria for CSD search).

To gain insight into the geometric changes experienced by the halogen-bonded components of (I_4_F_16_cb)·2(bpe), we constructed a novel *IsoStar* scatterplot (Bruno *et al.*, 1997 ▸) composed of all the structures in the Cambridge Structural Database (CSD, Version 5.38; Groom *et al.*, 2016 ▸), with N⋯I halogen bonds involving *p*-C_6_F_4_-I and 4-pyridyl groups (Fig. 4 ▸). A total of 116 halogen bonds from 83 reported crystal structures were analysed to study the positioning of the 4-pyridyl groups in relation to a best fit *p*-C_6_F_4_-I moiety. Each 4-pyridyl group of each halogen bond is populated into symmetrically equivalent quadrants, which reflects the symmetry of the *p*-C_6_F_4_-I group and defines a ‘window’ of experimental geometries of halogen bonds. The boundary of the window is defined according to conventional N⋯*X* distances and C—*X*⋯N angles (θ) used to describe halogen bonds. The boundary is rectangular in shape (see insets, Fig. 4 ▸), which reflects the directionality of the N⋯I force. We also took note of the π_centroid_—N⋯I angle (φ). Given that the separation distance of the C atoms of the C=C bonds decreases to form the C—C bonds, we expected a change in the π_centroid_—N⋯I angle to describe the ‘sweep’ of the 4-pyridyl group away from *ca* 180° in the photodimerization.

The *IsoStar* scatterplot analysis shows that the positions of the 4-pyridyl groups before and after the photodimerization lie within the window of geometries reported in the CSD (Fig. 4 ▸, wireframe). The ranges of the N⋯I bond distances and C—I⋯N angles from the CSD are 2.667–3.040 Å and 159.5–180.0°, respectively (see table in Fig. 2 ▸). The distances and angles for (I_4_F_16_cb)·2(bpe) and (I_4_F_16_cb)·2(tbcb) fall within these ranges. The π_centroid_—N⋯I angles from the CSD range from 132.6 to 180.0°. This range is larger than that of the C—I⋯N angles, whereas the corresponding angles for (I_4_F_16_cb)·2(bpe) and (I_4_F_16_cb)·2(tbcb) fall within the values. Considered together, we believe the *IsoStar* scatterplot shows that the changes in geometry of the 4-pyridyl groups in the photodimerization correspond to a linear-to-bent type of deformation from the plane of the best-fit *p*-C_6_F_4_-I group.

The changes in geometry of the halogen bonds in the SCSC transformation involve changes to the N⋯*X* distances and C—*X*⋯N angles. Specifically, *XB*1 underwent N⋯I lengthening from 2.782 (8) to 2.878 (5) Å (see table in Fig. 2 ▸). This change in distance was accompanied by a 2.3° increase in the C—I⋯N angle from 170.7 (3) to 173.0 (3)°. For *XB*2 (Fig. 4 ▸, see table in Fig. 2 ▸), the N⋯I distance (2.781 Å) remained largely intact while, in contrast to *XB*1, the C—I⋯N angle underwent a decrease of 1.7°. To accommodate the generation of the cyclo­butane, both pyridyl groups underwent movements corresponding to reductions in the π_centroid_—N⋯I angles of *XB*1 and *XB*2 from 172.6 (9) to 161.1 (2)° and from 175.8 (3) to 166.4 (2)°, respectively.

### 3.3. Single-crystal reactivity and movements of halogen-bonded components   

Metrangolo and Resnati (Caronna *et al.*, 2004 ▸) described the first example of a solid-state [2+2] photodimerization mediated by N⋯I halogen bonds (Scheme 2). A tetratopic molecule based on pentaerythritol (pethr) directed the formation of tpcb in a one-dimensional ribbon-type cocrystal assembly (Scheme 2); the solid-state reaction was studied in powder form. In later work, we showed that I_4_F_16_cb itself can be synthesized as a powdered cocrystal using dpn in a cyclo­addition sustained by N⋯I halogen bonds (Sinnwell & MacGillivray, 2016 ▸) (Scheme 3). For the case of (I_4_F_16_cb)·2(bpe), the SCSC reveals that the components of the N⋯I halogen bonds undergo linear-to-bent bending in order to accommodate the formation of tpcb in the solid state. We note that movements of components in the form of rotations of molecules engaged in N⋯*X* halogen bonds have been very recently considered as being relevant in the development of molecular-scale machine assemblies (Catalano *et al.*, 2015 ▸). *X*⋯*X* interactions (Mukherjee & Desiraju, 2014 ▸) have also been discussed as determinants in supporting the elasticity of molecular crystals that results in macroscopic changes to morphologies (Ghosh *et al.*, 2015 ▸; Saha & Desiraju, 2016 ▸). To the best of our knowledge, the role of N⋯I halogen bonds in supporting an SCSC photodimerization has not been reported. These observations are important as they suggest that halogen bonding may be employed in the pursuit of synthetic and materials applications of organic solids that exhibit SCSC photoreactivity and related single-crystal behavior.
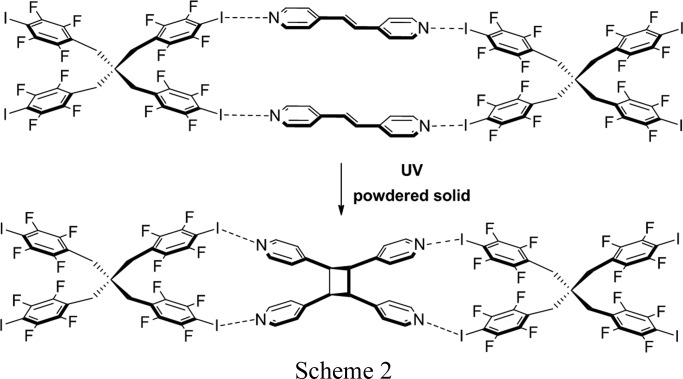
 
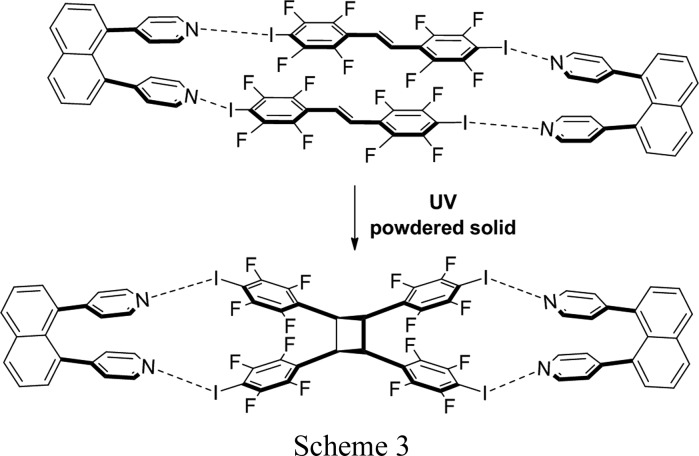


## 4. Conclusions   

We have demonstrated the ability of the components of N⋯I halogen bonds to support an SCSC [2+2] photodimerization. The SCSC transformation involves a linear-to-bent change in geometry of the halogen-bonded components. We believe this SCSC transformation provides insight into the dynamic processes of halogen-bonded components where achieving solids that exhibit fluidity, while maintaining structural integrity, can be important with implications in the design of complex materials and chemical systems (Barbour, 2006 ▸). Our focus now is to develop organic solids with components that assemble by a combination of noncovalent forces (*e.g.* hydrogen bonds and halogen bonds) and exhibit SCSC reactivity. We also envisage an ability to construct solids wherein halogen-bonded components exhibit a combination of movements (*e.g.* bending and rotation). Mechanical properties of the resulting materials will also be studied (Karunatilaka *et al.*, 2011 ▸).

## Supplementary Material

Crystal structure: contains datablock(s) publication_text, mcg164, mcg1611a. DOI: 10.1107/S2052252518007583/ed5014sup1.cif

Supporting information. DOI: 10.1107/S2052252518007583/ed5014sup2.pdf

CCDC references: 1521899, 1521900
