# Supplementary material for: Structural flexibility of halogen bonds showed in a single-crystal-to-single-crystal [2+2] photodimerization
Source: IUCrJ. 2018 Jun 22;5(Pt 4):491–6. doi: 10.1107/S2052252518007583 (PMC6038960; doi:10.1107/S2052252518007583)
Supplement: Supplementary file 2 [file m-05-00491-sup2.pdf]

IUCrJ

Volume 5 (2018)

Supporting information for article:

Structural flexibility of halogen bonds showed in a single-crystal-to-single-crystal [2+2] photodimerization

Michael A. Sinnwell, Jared N. Blad, Logan R. Thomas and Leonard R. MacGillivray

Supplementary information (SI)

Single-crystal-to-single-crystal [2+2] photodimerization mediated by the N···I halogen bond

S1. Syntheses

S2. ^{1}H NMR Spectral Data

S3. Powder and Single-Crystal X-ray Diffraction Data

S4. Halogen Bond Metrics

S5. CSD Data and IsoStar Video Files

S6. References

S1. Syntheses and Photoreactions

I_4F_{16}cb was synthesized according to the literature.[1] **bpe** (SIGMA) and solvents were commercially available and used without further purification (FISHER). Cocrystals of (**I_4F_{16}cb**)·2(**bpe**) as colorless plates were obtained *via* the addition of a solution of **I_4F_{16}cb** (23 mg, 0.02 mmol) in 1.5 mL of acetonitrile into a solution of **bpe** (11 mg, 0.04 mmol, 1:2 molar ratio) in 1.5 mL of acetonitrile. Single crystals suitable for single-crystal X-ray diffraction studies were obtained after one day. Photoreactions of (**I_4F_{16}cb**)·2(**bpe**) were conducted on a glass plate using UV-radiation from a 450W medium-pressure mercury lamp inside an ACE Glass photochemistry cabinet. ^{1}H NMR spectra were recorded using a Bruker AVANCE-300 NMR spectrometer operating at 300 MHz using DMSO-d_6 as NMR solvent. All data were processed with MestReNova suite of software programs.

S2. ^{1}H NMR Spectral Data.

^{1}H-NMR spectra were recorded using a Bruker AVANCE-300 NMR spectrometer operating at 300 MHz using DMSO-d_6 as NMR solvent. All data were processed with MestReNova 10.0 suite of software programs.

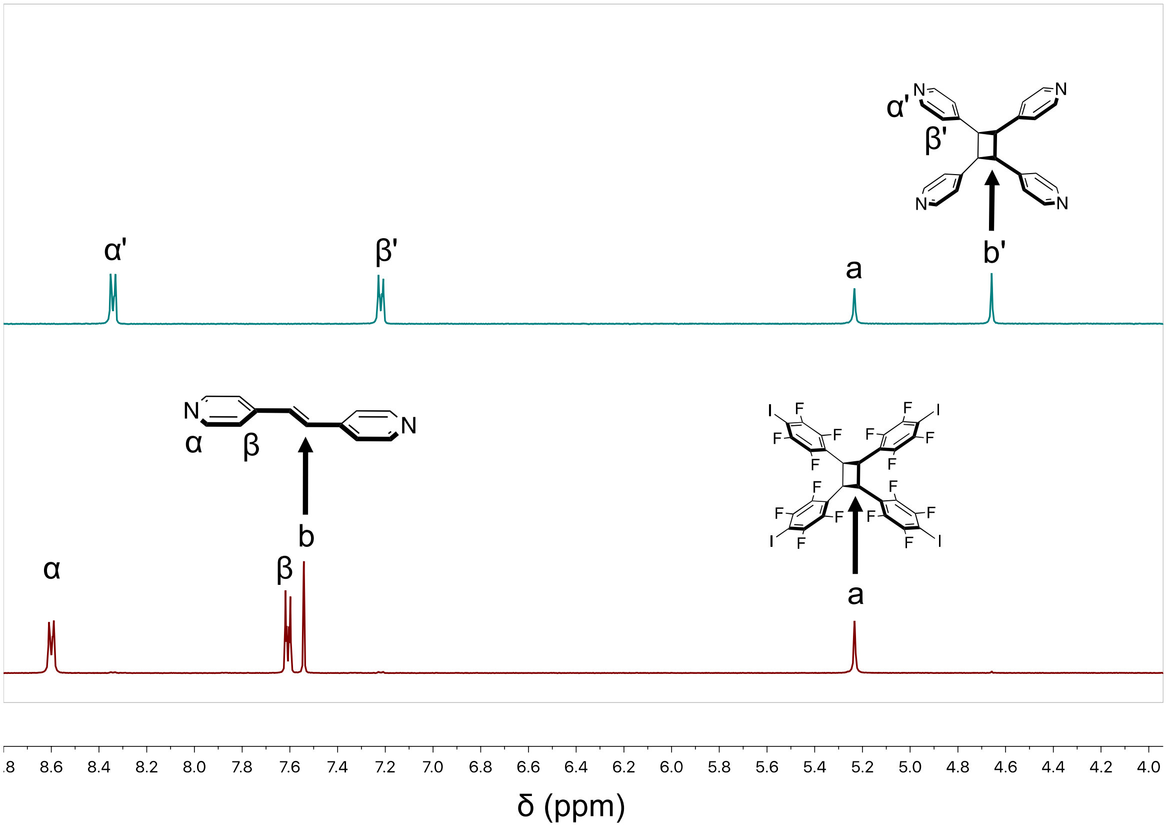

Figure S1. ^{1}H NMR spectra of (**I_4F_{16}cb**)·2(**bpe**) before UV irradiation (0 h) and (top) after UV irradiation (30 h) (DMSO-d_6).

S3. X-ray Diffraction Data

Powder X-ray Diffraction

PXRD data were collected from samples mounted on glass slides by a Siemens D5000 X-ray diffractometer using CuKα1 radiation (λ = 1.54056 Å) (scan type: locked coupled; scan mode: continuous; step size: 0.02°).

Single-crystal X-ray Diffraction

Single crystal data for (**I_4F_{16}cb**)·2(**bpe**) were collected with a Bruker APEX II Kappa diffractometer equipped with an Oxford Cryostream low temperature device using MoKa radiation (l = 0.71073 Å) after having been secured to Mitegen magnetic mounts using Paratone oil. Data collection strategies to ensure maximum data redundancy and completeness were calculated using Apex2.[2] All calculation dealing with data collection, initial indexing, frame integration, Lorentz- polarization corrections and final cell parameter were again carried out by Apex2.[2] Single-crystal diffraction data for (**I_4F_{16}cb**)·(**tpcb**) were collected on a Nonius Kappa CCD single-crystal X-ray diffractometer at room temperatures using MoKα radiation (λ = 0.71073 Å). Data collection, cell refinement, and data reduction were performed using Collect[3] and HKL Scalepack/Denzo,[4] respectively. All structures were solved via direct methods using ShelXT[5] and refined using ShelXL[5] in the Olex2[6] graphical user interface. All nonhydrogen atoms were identified from the difference Fourier map within several refinement steps. Hydrogen atoms associated with carbon atoms were refined in geometrically constrained positions with isotropic thermal parameter $U_{iso}(H) = 1.2\ U_{eq}(C_{CH})$.

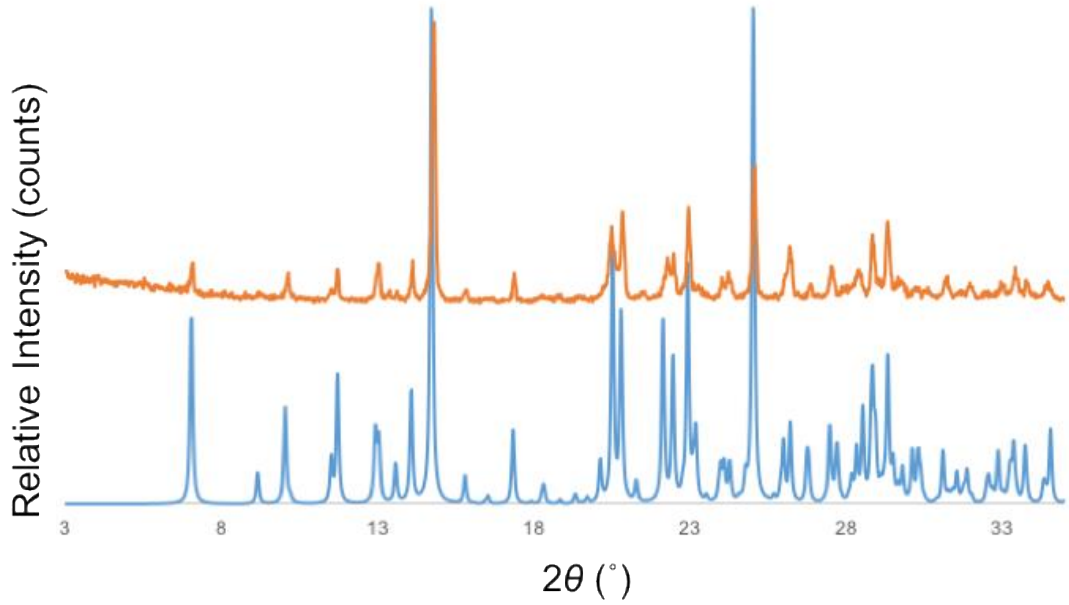

Figure S2. Powder X-ray diffraction of powder (**I_4F_{16}cb**)·2(**bpe**) (top) and simulated pattern from single-crystal X-ray diffraction data.

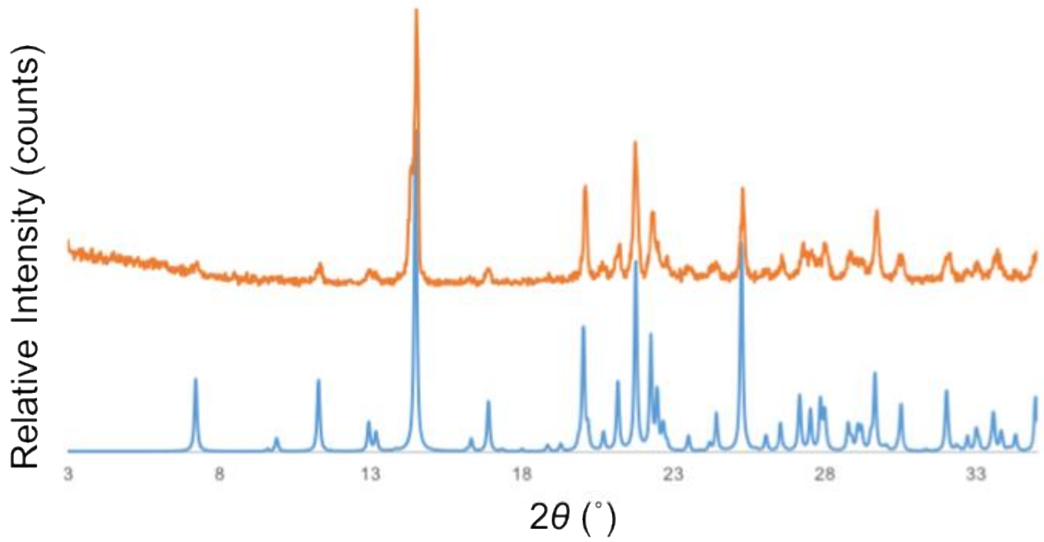

Figure S3. Powder X-ray diffraction of (**I_4F_{16}cb**)·(**tpcb**) (top) and simulated pattern from single crystal X-ray diffraction data.

Table S1. Crystal data and structure refinement for (**I_4F_{16}cb**)·2(**bpe**).

CCDC deposition number	1521899
Empirical formula	$C_{52}H_{24}F_{16}I_4N_4$
Formula weight	1516.35
Temperature/K	298.15
Crystal system	monoclinic
Space group	$P2_1/c$
a/Å	13.0184(13)
b/Å	9.7153(10)
c/Å	19.986(2)
α/°	90
β/°	104.649(5)
γ/°	90
Volume/Å^3	2445.6(4)
Z	2
ρ_{calc}g/cm^3	2.059
μ/mm^{-1}	2.653
F(000)	1440.0
Crystal size/mm^3	0.4 × 0.19 × 0.04
Radiation	MoKα (λ = 0.71073)
2Θ range for data collection/°	5.926 to 52.87
Index ranges	$-16 \leq h \leq 16$, $-12 \leq k \leq 11$, $-25 \leq l \leq 23$
Reflections collected	52897
Independent reflections	5012 [R_{int} = 0.0305, R_{sigma} = 0.0162]
Data/restraints/parameters	5012/704/639
Goodness-of-fit on F^2	1.085
Final R indexes [I>=2σ (I)]	R_1 = 0.0198, wR_2 = 0.0408
Final R indexes [all data]	R_1 = 0.0310, wR_2 = 0.0460
Largest diff. peak/hole / e Å^{-3}	0.45/-0.41

Table S2. Crystal data and structure refinement for (**I_4F_{16}cb**)·(**tpcb**).

CCDC deposition number	1521900
Empirical formula	$C_{52}H_{24}F_{16}I_4N_4$
Formula weight	1516.35
Temperature/K	298.15
Crystal system	monoclinic
Space group	$P2_1/c$
a/Å	12.9647(13)
b/Å	10.2219(10)
c/Å	19.519(10)
α/°	90
β/°	109.387(5)
γ/°	90
Volume/Å^3	2440.0(13)
Z	2
ρ_{calc}g/cm^3	2.064
μ/mm^{-1}	2.659
F(000)	1440.0
Crystal size/mm^3	0.14 × 0.12 × 0.05
Radiation	MoKα (λ = 0.71073)
2Θ range for data collection/°	5.956 to 50.786
Index ranges	$-11 \le h \le 15$, $-12 \le k \le 12$, $-23 \le l \le 23$
Reflections collected	12227
Independent reflections	4464 [R_{int} = 0.0670, R_{sigma} = 0.0580]
Data/restraints/parameters	4464/473/548
Goodness-of-fit on F^2	1.043
Final R indexes [I>=2σ (I)]	R_1 = 0.0330, wR_2 = 0.0548
Final R indexes [all data]	R_1 = 0.0626, wR_2 = 0.0627
Largest diff. peak/hole / e Å^{-3}	0.50/-0.52

S4. Halogen Bond Metrics

Table S3. Halogen-bond bending metrics: (**I_4F_{16}cb**)·2(**bpe**), (**I_4F_{16}cb**)·(**tpcb**), and CSD ranges.

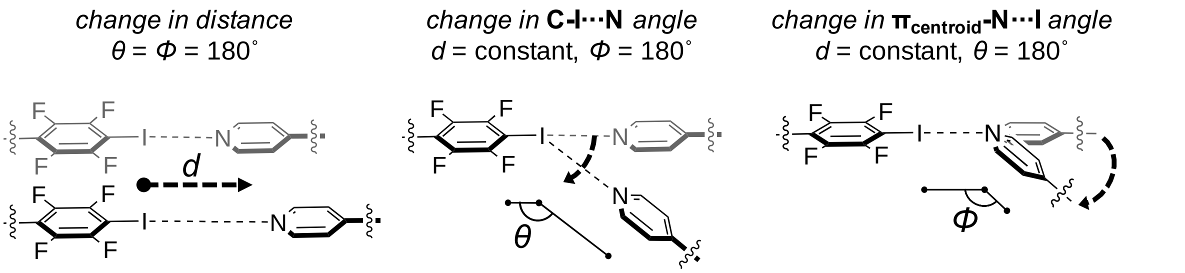

	N···I Distance (Å)		C-I···N (θ˚)		π centroid-N···I (Φ˚)	
	XB1	XB2	XB1	XB2	XB1	XB2
before: (**I_4F_{16}cb**)·2(**bpe**)	2.782(8)	2.781(8)	170.7(3)	173.7(4)	172.6(9)	175.8(3)
	Δ 26%	Δ 0%	Δ 11%	Δ -8.3%	Δ -24%	Δ -20%
after: (**I_4F_{16}cb**)·(**tpcb**)	2.878(5)	2.781(5)	173.0(3)	172.0(5)	161.1(2)	166.4(2)
CSD ranges:	2.667 - 3.040		159.5 - 180.0		132.6 - 180.0	
Δ_{range}	0.373		20.5		47.4	
mean	2.825		175.0		169.7	

Table S4. Halogen Bond Metrics

	halogen bond	N···I Distance (Å)	C-I···N (°)	$\pi_{centroid}$-N···I (°)
(I_4F_{16}**cb**)·2(**bpe**)	C1A-I1A···N1A-$\pi_{centroid}$	2.782(8)	170.7(3)	172.6(9)
	C1A-I1A···N1B-$\pi_{centroid}$	2.80(3)	167(1)	167.1(2)
	C1B-I1B···N1A-$\pi_{centroid}$	2.821(9)	164.6(4)	173.0(8)
	C1B-I1B···N1B-$\pi_{centroid}$	2.84(3)	161(1)	161.9(2)
	C12A-I2A···N2A-$\pi_{centroid}$	2.781(8)	173.7(4)	175.8(3)
	C12A-I2A···N2B-$\pi_{centroid}$	2.80(2)	178.3(9)	162.6(1)
	C12B-I2B···N2A-$\pi_{centroid}$	2.908(9)	171.3(5)	176.7(5)
	C12B-I2B···N2B-$\pi_{centroid}$	2.94(2)	166.3(7)	160.1(1)
(I_4F_{16}**cb**)·(**tpcb**)	C1A-I1A···N1-$\pi_{centroid}$	2.878(5)	173.0(3)	161.1(2)
	C1B-I1B···N1-$\pi_{centroid}$	2.84(1)	177(1)	158.9(4)
	C12A-I2A···N2-$\pi_{centroid}$	2.781(5)	172.0(5)	166.4(2)
	C12B-I2B···N2-$\pi_{centroid}$	2.80(1)	166(1)	167.4(4)

S5. CSD Data and IsoStar Video Files

IsoStar Video Files

Video S1. IsoStar scatterplot of structures from the CSD (wireframe) and **XB1** from (**I_4F_{16}cb**)·2(**bpe**) (gray) and (**I_4F_{16}cb**)·(**tpcb**) (black). Highest occupied **XB1** in one quadrant.

Video S2. IsoStar scatterplot of structures from the CSD (wireframe) and **XB2** from (**I_4F_{16}cb**)·2(**bpe**) (gray) and (**I_4F_{16}cb**)·(**tpcb**) (black). Highest occupied **XB2** in one quadrant.

CSD data

Search Overview

Database(s):	CSD version 5.38 updates (Nov 2016)<br>CSD version 5.38 (November 2016)<br>CSD version 5.38 (November 2016)	
Restriction Info:	No refcode restrictions applied	
Filters:	3D coordinates determined<br>No errors<br>No ions<br>Only Organics	R factor <= 0.075<br>Not polymeric<br>No powder structures
Percentage Completed:	100%	

Single query used. Search found structures that:

match

Query 1

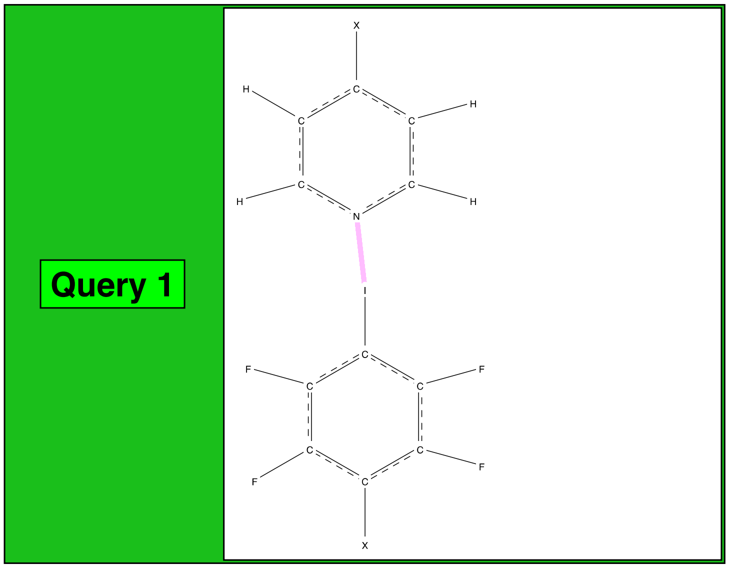

S6. References

1. Sinnwell, M. A. & MacGillivray, L. R. (2016). *Angew. Chem. Int. Ed.* **55**, 3477-3480.

2. Bruker (2012). *APEX2*. Bruker AXS Inc., Madison, Wisconsin, USA.

3. Hooft R. W. W., *COLLECT*. Nonius BV, Delft, The Netherlands, (1998).

4. Otwinowski, Z. & Minor, W. (1997). Methods in Enzymology, Vol. 276, Macromolecular Crystallography, Part A, edited by C. W. Carter Jr & R. M. Sweet, pp. 307-326. New York: Academic Press.

5. Sheldrick, G. M. (2015). Acta Cryst. A71, 3-8.

6. Dolomanov, O. V., Bourhis, L. J., Gildea, R. J., Howard, J. A. K. & Puschmann, H. (2009). *J. Appl. Crystallogr.* **42**, 339-341.
